# Psoriasis and Fibromyalgia: A Systematic Review

**DOI:** 10.3390/jpm14020165

**Published:** 2024-01-31

**Authors:** Martina D’Onghia, Francesco Ursini, Elisa Cinotti, Laura Calabrese, Linda Tognetti, Alessandra Cartocci, Laura Lazzeri, Bruno Frediani, Pietro Rubegni, Emanuele Trovato

**Affiliations:** 1 Department of Medical, Surgical and Neurological Sciences, Dermatology Section, University of Siena, 53100 Siena, Italy; m.donghia@student.unisi.it (M.D.); laura.calabrese@unisi.it (L.C.); linda.tognetti@dbm.unisi.it (L.T.); alessandra.cartocci@dbm.unisi.it (A.C.); laura.lazzeri@ao-siena.toscana.it (L.L.); pietro.rubegni@unisi.it (P.R.);; 2 Medicine and Rheumatology Unit, IRCCS Istituto Ortopedico Rizzoli, 40136 Bologna, Italy; francesco.ursini2@unibo.it; 3 Department of Biomedical and Neuromotor Sciences (DIBINEM), University of Bologna, 40126 Bologna, Italy; 4 Rheumatology Unit, Department of Medicine, Surgery and Neurosciences, University of Siena, 53100 Siena, Italy; bruno.frediani@unisi.it

**Keywords:** psoriasis, fibromyalgia, systematic review

## Abstract

Psoriasis is a chronic inflammatory cutaneous condition characterized by several comorbidities, including musculoskeletal disorders. While the association with psoriatic arthritis has been widely addressed in literature, the aim of the present systematic review was to identify all available evidence on the relationship between psoriasis and fibromyalgia, a musculoskeletal syndrome primarily characterized by chronic widespread pain. We followed the Preferred Reporting Items for Systematic Reviews and Meta-Analyses (PRISMA) guidelines, and MedLine and Web of Science (WOS) databases were searched for literature up to March 2023. After the removal of duplicate records, a total of 11 articles were deemed eligible for inclusion in a qualitative synthesis. Our results suggested that psoriatic patients had a higher prevalence of fibromyalgia (8–30%), with a very high impact on symptoms of psoriasis. Moreover, fibromyalgic patients had a slightly increased prevalence of psoriasis (2.2–6.7%) compared to the control groups. Finally, several studies demonstrated the substantial impact of fibromyalgia on psoriatic outcome measures in patients with concomitant psoriatic arthritis. In conclusion, available data support a potential interplay between psoriasis and fibromyalgia, but further research is encouraged in this area.

## 1. Introduction

Fibromyalgia (FM) is a chronic syndrome primarily characterized by widespread pain, fatigue, and sleep disturbances but also by a variety of associated symptoms, including cognitive impairment, anxiety, and depression [1]. Fibromyalgia mainly affects women over 50 years old, reaching an estimated global prevalence of 2% to 3% [2]. The severe symptom burden of FM negatively influences patients’ social functioning and quality of life, resulting in high direct and indirect costs [3]. 

The real nature of FM is still controversial. Pathogenesis encompasses both central and peripheral mechanisms involved in pain processing through a complex interplay between genetic, immunological, inflammatory, endocrine, social, and cognitive components [4]. 

Although FM is recognized as a distinct condition, a growing body of literature has focused on the association between FM and other diseases not necessarily defined by chronic pain, such as neurological, gastrointestinal, and mental health illnesses [5]. Within this context, recognizing FM comorbidity is crucial due to its impact on overall self-perceived health status: indeed, a misdiagnosis could result in inaccurate assessment of the severity of the primary disease (often overestimated), which may impact how the underlying condition is managed and treated. 

Compared to the general population, FM is particularly common in chronic inflammatory arthropathies, including psoriatic arthritis (PsA) [6], a complex inflammatory musculoskeletal disease with the highest prevalence among 30–60-year-old patients and with no sex differences [7]. PsA may occur in around ~30% of patients with skin psoriasis (PsO) [7], and it is also associated with higher risk of developing comorbidities, including uveitis, inflammatory bowel disease, and metabolic syndrome, with a significant impact on quality of life [8]. Although the precise mechanisms underlying PsA pathogenesis are poorly understood, several immune-inflammatory pathways have been identified, leading to the development of novel therapeutic strategies [9]. However, a sizable portion of PsA patients still report unsatisfactory results following such treatments and experience residual pain and fatigue [10]. Given some clinical similarities between these conditions, it can be difficult to differentiate PsA-associated polyenthesitis from concurrent FM, and it is yet to be quantified how much of treatment failure in PsA patients may be attributed to concurrent FM [11]. Furthermore, while the relationship between FM and rheumatic disorders has received considerable interest, little is known about the association between FM and PsO, although—beyond joint complaints—many FM symptoms are also frequent in PsO, such as fatigue [12], sleep disturbances [13], and cognitive impairment [14].

Despite several comorbidities that have been reported in FM patients, this condition is not classically considered to be associated with cutaneous findings, and the available literature on this topic is still limited. However, current evidence has shown objective differences in the skin biopsies of FM patients compared with healthy subjects [15]. Particularly, multiple studies have described increased mast cell and inflammatory cytokines counts [16,17], altered collagen metabolism [18], cutaneous microcirculatory changes, autonomic nervous system dysfunction, and increased cutaneous opioid receptors in the skin of FM patients [16]. It is well accepted that the impact of PsO, a chronic immune-mediated inflammatory disease, extends far beyond the skin. Psoriasis affects approximately 125 million people worldwide, with a mean age of onset between 30 and 50 years [19], representing a huge health problem for patients and society [20]. The pathogenesis of psoriasis is multifaceted, resulting from the interaction between environmental, genetic, and immunologic factors [21]. The constellation of systemic-associated comorbidities, including cardiometabolic, gastrointestinal, kidney diseases, malignancy, infection, and psychiatric disorders, highlights the importance of correct management of those patients, which is often challenging [22,23]. 

On this background, the aim of this systematic review was to identify all available data on the prevalence of FM in PsO patients and to synthetize what is known regarding the relationship between PsO severity and FM.

## 2. Materials and Methods

### 2.1. Search Strategy

MedLine (via PubMed), Web of Science (WOS). And Cochrane Central Register of Controlled Trials (CENTRAL) databases were searched up to 10 March 2023. The main search in MedLine was performed using the string “(fibromyalgia” OR “chronic fatigue syndrome” OR “chronic fatigue disorder”) AND (“psoriasis” OR “psoriatic arthritis” OR “spondylarth*”)”, while the main search in CENTRAL was performed using the string “(“fibromyalgia” OR “chronic fatigue syndrome” OR “chronic fatigue disorder”) AND (“psoriasis” OR “psoriatic arthritis” OR “spondyloarthritis”)”. Finally, the main search in WOS was performed using the string “[TS= ((“fibromyalgia” OR “chronic fatigue syndrome” OR “chronic fatigue disorder”) AND (“psoriasis” OR “psoriatic arthritis” OR “spondylarth*”))]”. Additionally, relevant keywords were used in different combinations for free-hand search, and the bibliography of selected articles was reviewed. The decision to include the keywords “chronic fatigue syndrome” was adopted to increase the sensitivity of the search strategy (e.g., identification of studies centered on chronic fatigue syndrome (CFS) but also including individuals satisfying criteria for FM, given the clinical overlap between the two clinical entities), although the focus of our article was FM. A similar rationale was used for including the keywords “psoriatic arthritis” and “spondyloarthritis”.

The search was designed and performed by one author (MD) under the supervision of a senior investigator (FU). No date restriction was applied. We followed the Preferred Reporting Items for Systematic Reviews and Meta-Analyses (PRISMA) guidelines [24] for preparing our manuscript.

### 2.2. Eligibility Criteria

For the purpose of this study, only articles written in the English language were considered eligible. To be included in the final review, studies had to be published as full-text original articles in international, peer-reviewed journals. Randomized controlled trials (RCT), quasi-RCT (trials in which allocation to treatment was made by alternation, use of alternate medical records, date of birth, or other expected methods), prospective or retrospective cohort studies, and cross-sectional studies were deemed eligible. The population, intervention, comparator, outcome (PICO) framework was used to build the search question. All studies meeting the following criteria were included in the final review:

- Population: adult patients diagnosed with FM or PsO;
- Intervention: not applicable;
- Comparison: healthy controls or no control group;
- Outcome:
  - Studies assessing the prevalence of PsO in FM patients.
  - Studies assessing the prevalence of FM in PsO patients.
  - Studies assessing the association between severity of FM clinical features (composite measures of symptoms severity, pain, tender point count, stiffness, fatigue, physical functioning, sleep, depression, anxiety, cognitive dysfunction, QoL) and outcomes measures in PsO and vice versa.

Studies focused on PsA only, without adequate subanalysis to extrapolate information related to cutaneous domain, were excluded.

### 2.3. Study Selection Process and Data Extraction

**Figure 1:**
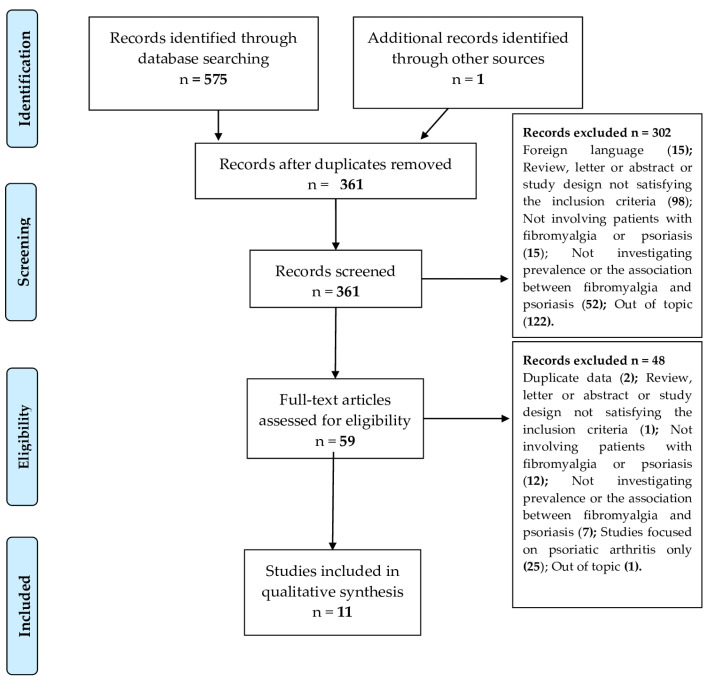
PRISMA—compliant study selection flow chart.

After removal of duplicate records, three reviewers (MD, ET, and LC) independently screened titles and abstracts for the first-step evaluation. Following the screening phase, two reviewers (MD and ET) independently evaluated the full text of the remaining articles to determine eligibility for inclusion in the final review. Disagreements among the reviewers were resolved by discussion with a third senior investigator (FU) until reaching a final consensus. A detailed flowchart of the study selection process is reported in Figure 1. Additional studies were identified using bibliographical information of relevant articles.

### 2.4. Quality Assessment 

The quality of studies was evaluated using the Newcastle-Ottawa Quality Assessment Scale (NOS) [25], extensively used to assess the risk of bias in cohort and cross-sectional studies (Table 1). 

## 3. Results

### 3.1. Results of the Systematic Search

The search strategy identified 254 records in PubMed, 278 in WOS, and 43 in CENTRAL. In addition, a manual search identified one relevant article. After the removal of duplicates, a total of 361 studies proceeded to review. Of those, 302 articles were excluded following screening of titles and abstracts, and full text evaluation was performed on 59 articles. A total of eleven articles were deemed eligible for inclusion in qualitative synthesis, including ten cross-sectional studies and one longitudinal cohort studies (Figure 1). Quality assessment, performed using the NOS scale, showed that the overall risk of bias was high, since none of the included studies scored > 7, widely used as a cut-off for defining high quality (Table 1).

### 3.2. FM in PsO Patients: Higher Prevalence and Impact on Outcome Measures 

Two of the selected articles reported the prevalence of FM in PsO patients and the aftermath of comorbid FM on PsO disease activity [33,35] (see Table 2). 

Thune et al. [35] investigated the consequences of FM in a cross-sectional study of 1269 consecutive PsO patients. Among them, 230 (14.1%) had musculoskeletal pain not fulfilling the American College of Rheumatology (ACR) 1990 criteria for FM [37], while 105 (8.3%), mostly women (97.6%), fulfilled the criteria. Of note, 9% of patients had chronic widespread pain (CWP) not satisfying the 1990 FM criteria because of an insufficient tender point count. However, it is possible to anticipate that some of these patients may fulfil the 2016 ACR criteria that abolished the tender point count favoring subjective reporting of widespread pain. 

Although psoriasis extent did not differ between patients with and without FM, the two groups differed in terms of disease locations and phenotype. Specifically, the involvement of the head and palmoplantar pustulosis were more frequent in FM patients, while small lesions of the trunk and extremities were more common in individuals not satisfying FM criteria. No correlation was found between FM symptoms and extent of PsO (R < 0.25). On the other hand, tender point count, visual analogue scale (VAS) pain score, prevalence of sleep disturbances, morning tenderness, and daytime fatigue were higher in FM + PsO patients when compared to non-FM (all *p* < 0.05). In total, 27 patients (28.2%) with FM were diagnosed with PsA and 16 patients with possible PsA. By comparison, only 44/230 patients (19%) with musculoskeletal complaints but without FM were diagnosed as having PsA. 

In 2020, Mathkhor et al. [33] published the results of a cross-sectional study on 70 PsO patients and 70 age- and sex-matched controls from the general population. Patients with a diagnosis of PsA were excluded from the study. The overall prevalence of CWP not fulfilling the FM criteria in PsO patients was 52.9% (*n* = 37), while the overall prevalence of FM in PsO was 30% (*n* = 21), predominantly women (85.7%, *p* < 0.05). Psoriasis area severity index (PASI) score was higher in PsO + FM patients when compared to non-FM (respectively 57.9 ± 4.6 vs. 15.5 ± 4.6, *p* < 0.05). Lastly, FM-allied symptoms, such as morning stiffness, sleep disturbances, anxiety, fatigue, headache, and irritable bowel syndrome, were higher in PsO individuals when compared to the control group (*p* < 0.05). 

### 3.3. PsO in FM Patients: A Controversial Association 

In 2014 Laniosz et al. [32] performed a cross-sectional study to investigate the prevalence of dermatologic diseases and skin-related symptoms in 897 patients with a confirmed diagnosis of FM, reporting an overall prevalence of PsO of 2.2%, a figure that seems comparable to what is observed in the Western general population [38]. 

Erdogan et al. [29] in their study evaluated skin findings and skin-related symptoms in 105 female FM patients and 105 matched controls. From their analysis, PsO did not appear to be overrepresented in the FM population compared to the control group (*p* = 0.498). 

Finally, Kridin et al. [26] conducted a population-based study from a large database from Israel. A total of 18,598 FM patients were enrolled as cases, and age- and gender-matched randomly to controls without FM (*n* = 36,985). The prevalence of PsO was higher in FM patients compared to control subjects (6.7% vs. 4.8%, respectively; OR, 1.4; 95% C.I.: 1.3–1.5; *p* < 0.001) with the greatest association seen in patients between 30 and 49 years (1.5; 95% CI: 1.3–1.8; *p* < 0.001) and males (OR: 1.8; 95% C.I.: 1.4–2.3; *p* < 0.001).

This association was robust to a multivariate analysis accounting for age, gender, ancestry, socioeconomic status, and healthcare utilization (OR, 1.3; 95% C.I.: 1.2–1.4; *p* < 0.001). Finally, patients with coexisting FM and PsO presented with a significantly older age, a higher mean body mass index (BMI), and frequency of smoking when compared to FM only patients.

### 3.4. FM in PsA Patients: Results on PsO Outcome Measures 

Two of the reviewed studies demonstrated that PASI score was statistically higher in patients with concomitant FM and PsA [28,30]. In a cross-sectional study by Elsawy et al. [28], patients with PsA and FM had higher median PASI levels compared to PsA only [14.4 (0.9–49.6) vs. 8.3 (0.0–45.7), *p* = 0.043]; furthermore, there was a positive correlation between the Fibromyalgia Impact Questionnaire (FIQ) and PASI (R = 0.488, *p* = 0.018). Median Dermatology Life Quality Index (DLQI) was numerically higher but did not reach the level of statistical significance [13 (3–24) vs. 9 (0–30), *p* = 0.073]. Similarly, Iannone et al. [30] confirmed a higher PASI score in patients with coexisting PsA + FM compared to PsA only [0.2 (0.5) 0.4 vs. (1.4) 0.6, *p* = 0.0006], but no significant differences were observed with regard to onychopathy.

On the other hand, Brinkman et al. [27] did not find any significant difference in PASI score comparing PsA + FM versus PsA only patients [3 (0.8–8.6) vs. 2.1 (0.2–6.3), *p* = 0.22]. 

Again, Kancharla et al. [31] did not find any significant difference regarding age at onset, duration, or severity of PsO, evaluated through Body Surface Area (BSA) score or PASI, comparing PsA patients with and without FM; in univariate logistic regression, PASI was not associated with the risk of having comorbid FM [OR: 1.01 (0.88–1.15), *p* = 0.90] and no correlation of either FM Symptom Severity Scale (SSS), Widespread Pain Index (WPI), or FIQ was noted with severity of skin involvement measured by PASI or the duration of skin disease.

Furthermore, Ulus et al. [36] pointed out how psoriasis duration was higher among PsA + FM when compared to only PsA patients; however, Polachek et al. [34] did not find any difference in PsO duration between PsA patients with and without FM.

## 4. Discussion

Psoriasis is a chronic skin disease with a profound impact on patients’ well-being and quality of life, comparable to that observed in other chronic diseases such as cardiovascular diseases, diabetes, end-stage renal diseases, or cancer [39]. Furthermore, the disease burden of PsO is worsened by a higher risk of comorbidities such as arthritis, obesity, cardiometabolic derangement, psychiatric illness, and malignancy [40]. 

Although it is somewhat intuitive that rheumatological complaints in PsO patients can often be attributed to coexistent PsA, musculoskeletal pain is highly prevalent in PsO patients even in the absence of clinical, serological, and imaging features suggestive of PsA. A Danish population-based survey reported a prevalence of musculoskeletal pain of 68.3%, in contrast with the prevalence of PsA that was only about 20% [41].

This discrepancy may represent a limit in sensitivity to demonstrate subtlest inflammatory abnormalities—especially at the enthesis—but it is also possible to speculate that a link exists between PsO and other musculoskeletal disorders, including FM. 

Fibromyalgia, on the other hand, is a well-established aggravating factor in rheumatic diseases, and its misrecognition may lead to important consequences, in particular overinvestigation and overtreatment [42]. 

Unfortunately, the results of our systematic review clearly demonstrate that this topic has been poorly explored. Indeed, while several articles describe the association between PsA and FM (outside the scope of this review), little is known about the relationship between pure skin PsO and FM. 

Overall, we found only two studies reporting data on the prevalence of FM in PsO, suggesting that it is higher (8–30%) than that observed in the general population [33,35]. Despite this, we feel that comorbid FM can, at least in part, fill the gap between the highly reported musculoskeletal pain and the true prevalence of PsA in PsO patients. 

Although the putative pathways connecting PsO to FM are still unknown, some speculative hypotheses can be made based on available data. Despite the frequent consideration of physicians that psoriasis is not particularly associated with skin symptoms, cutaneous pain is reported by up to 42% of patients with PsO [43,44], and it is associated with several subjective unpleasant feelings such as pruritus, unpleasantness, aches, sensitivity, heat/burning, tenderness, and cramping. Specifically, PsO patients describe their pain as a stinging, hot, burning pain, suggesting a neurogenic origin, even though this topic has not been widely investigated in literature [45]. 

In this context, chronic nociceptive skin stimulation may subsequently lead to central sensitization [46], a mechanism supposed to underly FM associated with other painful diseases such as osteoarthritis [47]. Another possible explanation for the increased prevalence of FM in PsO may lie in the common underlying pathogenetic pathways associated with dysfunctional neurotransmitter systems, particularly the increased level of substance P [48,49]. Furthermore, inflammatory mechanisms are emerging as potential contributors to FM pathogenesis [50]. Interestingly, the distinctive signature of FM includes pro-inflammatory cytokines, which also play a role in the pathogenesis of PsO [51,52]. The results of several studies have shown the presence of increased levels of IL6 [53], IL8 [54], [55] and TNF-α [53,56] in FM patients, giving additional support to this linkage. Notably, Pernambuco et al. [57] found higher levels of IL17A in FM patients, which represents one of the major immunologic cytokines that drives the psoriatic disease [58]. Overall, the imbalance between pro-inflammatory and anti-inflammatory cytokines could underlie a common pathogenetic pathway associated with both FM and psoriatic disorders by contributing to the induction and maintenance of pain [59]. 

In addition to the increased number of inflammatory cytokines, a statistically significant increase in the number of mast cells, as well as increased mast cell degranulation have been shown in the skin of FM patients [16]. All of these processes have been shown to lead to increased pruritus, burning, and other forms of neuropathic discomfort. 

Other possible puzzle pieces can be found in the literature, such as the widespread occurrence of FM-allied symptoms in PsO patients.

Fatigue is a crucial symptom of FM [60] and there is a large clinical overlap between FM and chronic fatigue syndrome (CFS) [61]. Fatigue, on the other hand, is reported frequently (nearly 50% in [12]) in PsO patients, and PsO itself is associated with a 48% increase of subsequent development of CFS [62]. Although the mechanisms leading to fatigue in PsO are far from being ascertained, crucial molecular and cellular players of PsO immunopathogenesis [63,64] are believed to contribute by exploiting both central and peripheral mechanisms. 

Sleep disturbances affect more than 90% of patients with FM [65]; similarly, the prevalence of insomnia in PsO has been reported in 5.9–44.8% of patients, while the prevalence of OSA and restless leg syndrome (RLS) ranged from 36–82% and 6–15%, respectively [66].

Further, FM and PsO share an increased risk of psychiatric diseases. The bidirectional association between FM and depression or anxiety has been widely demonstrated in literature [67], as the role of depression in PsO is well known, traditionally explained as a response to psychosocial factors and impaired quality of life, even though a recent hypothesis evoked the dysregulation of common inflammatory and immunological pathways in depression and PsO [68]. Intriguingly, a study by Pinho de Oliveira Ribeiro et al. [69] suggested that DMARDs, such as methotrexate, used to treat severe PsO and PsA, conferred the highest risk of depression, anxiety, and suicidal ideation. On this basis, since FM and PsO are individually related to psychosocial disability, the coexistence of both may worsen depression, influencing management and treatment of those patients and increasing the risk of drug interaction and adverse effects, in a vicious circle. 

Another intriguing link between those two conditions may be represented by obesity. It is known that obesity has a cardinal role in pathogenesis of skin diseases, including PsO, by producing adipokines, a group of proinflammatory cytokines involved in immunity and responsible for a chronic low-grade inflammatory state [70]. The overexpression of leptin in obese patients has been shown to reduce T regulatory cells (T-regs) and amplify the local inflammation, as reflected by higher IL-17 and IL-23 in obese subjects compared to controls. Moreover, IL-6 can break homeostasis in immune tolerance by inhibiting TGFβ-driven T-reg differentiation and promoting Th17 differentiation [71,72]. Based on the current knowledge, FM and obesity share common pathophysiological mechanisms, in which obesity seems to represent an aggravating factor and a potential trigger in FM patients, while FM may favor or worsen the development of obesity. Another possible hypothesis is that obesity may “trigger” FM symptoms by fueling the low-grade inflammatory background [73]. Accordingly, the effect of therapeutic weight loss may provide potential benefit in the comprehensive treatment of both PsO and FM patients.

From an epidemiological point of view, data indicate that the occurrence of both FM and PsO varies in relation to demographic characteristics, such as age, gender, and geographic region, but also according to the diagnostic criteria used to define the primary condition [2,74,75]. We found that FM patients with PsO were mostly middle-aged women, which is not particularly surprising since FM has a strong female predominance [26,29,32]. Conversely, there is lack of agreement on specific gender differences in PsO, and a slight male predominance was reported in recent studies [76]. Nevertheless, our results showed that FM was more frequent in female patients with PsO [33,35], suggesting a possible differential gender distribution of FM in the setting of this cutaneous disease. However, further research is required to determine the underlying mechanisms to explain this relationship. 

Our review attempted to shed light on the relationship between FM and PsO and opens many new avenues for research, but despite its potential, some limitations must be acknowledged. First, literature on skin findings in FM patients is still lacking, and the few available studies are characterized by high heterogeneity regarding design, sample size, adequate age- and gender-matched control group, demographic characteristics of patients (such as gender ratio), severity of PsO, and classification criteria used. Second, in some studies, the presence of FM was defined using the 1990 ACR criteria, based on tender point count; however, a large overlap between FM tender points and potential enthesitis sites is now recognized that can lead to misclassification [77,78,79]. 

Finally, most of the eligible studies had a cross-sectional design; hence, distinction between higher PsO disease activity precipitating secondary FM versus comorbid FM inflating PsO disease activity cannot be ascertained.

## 5. Conclusions

In conclusion, our review suggests that a relationship between Pso and FM is likely to exist, although the mechanisms are far from being clear. Comorbid FM can represent an aggravating factor reverberating on some features of PsO and an amplifier of pain, fatigue, and physical disability, leading to poor QoL independently from the primary skin disease. For these reasons, to achieve global well-being, it seems important to evaluate the presence of comorbid FM, so that the clinicians can make appropriate decisions on the management and treatment of the underlying condition, as well as instituting nonpharmacological or pharmacologic treatment for FM. Future studies are mandatory to shed light on causative mechanisms beyond the possible association between these disorders. 

## Figures and Tables

**Table 1:** Quality assessment of included studies according to the Newcastle—Ottawa Scale (NOS).

Author, Year	Item S1	Item S2	Item S3	Item S4	Item C1	Item E1	Item E2	Item E3	Score
Kridin, 2020 [26]	b *	b	a *	a *	a *	d	a *	c	******
Brinkman, 2016 [27]	a *	a *	c	c	c	b *	c	c	***
Elsawy, 2021 [28]	a *	a *	c	c	c	b *	c	d	***
Erdogan, 2016 [29]	a *	a *	a *	a *	a *	c	a *	c	******
Iannone, 2020 [30]	b *	c	c	c	c	a *	a *	d	***
Kancharla, 2022 [31]	a *	a *	c	c	c	b *	c	d	***
Laniosz, 2014 [32]	b	b	c	c	c	d	c	c	-
Mathkhor, 2020 [33]	a *	b	a *	a *	a *	c	a *	c	*****
Polachek, 2021 [34]	a *	a *	c	c	c	b *	c	c	***
Thune, 2005 [35]	a *	a *	c	c	c	b *	c	d	***
Ulus, 2019 [36]	a *	b	a *	b	a *	c	a *	c	****

Legend: C, Comparability; E, Exposure; S, Selection. The stars (*) obtained by each article are summed up to obtain a final score, representing the overall quality of the study, reported in the last column.

**Table 2:** Summary of systematic review assessing the association between fibromyalgia and psoriasis.

Author, Year	Country	Recruitment	Year	Patients (% F)	Patients Age, Years, Mean (SD)	Controls (%F)	Design	FM Criteria	PsO or PsA Criteria
Thune, 2005 [35]	Norway	Consecutive outpatient	1997–2000	1269 (56) PsO patients	49.5 (12.8)	NR	Cross sectional	ACR 1990	Clinical examination
Laniosz, 2014 [32]	USA	Outpatient	2008	845 (90.6) FM patients	48.9	NR	Cross sectional	ACR 1990/ACR 2010/ACR 2011	Clinical examination
Brinkman, 2016 [27]	Israel	Consecutive outpatient	2013–2014	73 (57.7) PsA patients	51.7 (13.5)	NR	Cross sectional	ACR 2010/ACR 1990	CASPAR
Erdogan, 2016 [29]	Turkey	Inpatient	NR	105(100) FM patients	45.5 (8.7)	105(100)	Cross sectional	ACR 2010	Dermatological examination
Ulus, 2019 [36]	Turkey	Inpatient	2017–2018	50 (72) PsA patients	47 (23–64) *	NR	Cross sectional	CASPAR	ACR 2010
Iannone, 2020 [30]	Italy	Outpatient	2010–2017	238 (53) PsA patients	49.2 (12)	NR	Cohort study	CASPAR	ACR 2010/ACR1990
Kridin, 2020 [26]	Israel	Health Care Database	2017	18,598 (91) FM patients	56.5 (14.1)	36,985 (91)	Cross sectional	EHR	EHR
Mathkhor, 2020 [33]	Iraq	Outpatient and inpatient	2018–2020	70 (57) PsO patients	50.7 (7.3)	70 (57)	Cross sectional	ACR 1990	Clinical examination
Elsawy, 2021 [28]	Egypt	Outpatient consecutive	2017–2018	60 (43.3) PsA patients	49.30 (11.69)	NR	Cross sectional	CASPAR	ACR 2016
Polacheck, 2021 [34]	Israel	Consecutive outpatient	2018–2020	156 (56) PsA patients	52.7 (12.7)	NR	Cross sectional	CASPAR	ACR 2016
Kancharla, 2022 [31]	India	Consecutive outpatient	2014–2015	102 (50) PsA patients	43.4 (12.4)	NR	Cross sectional	CASPAR	ACR 2010

* age expressed as median (minimum-maximum). Legend: ACR, American College of Rheumatology; CASPAR, ClASsification criteria for Psoriatic Arthritis; EHR, electronic health record; FM, fibromyalgia; NR, non-reported; PsA, Psoriatic arthritis; PsO, Psoriasis.

## Data Availability

Data are available upon request to the corresponding author.
